# Editorial: Exploring genetic and environmental factors in skeletal muscle development

**DOI:** 10.3389/fvets.2025.1636652

**Published:** 2025-08-11

**Authors:** Jia Luo, JingJing Du

**Affiliations:** ^1^Southwest University, Chongqing, China; ^2^Max Planck Institute for Heart and Lund Research, Bad Nauheim, Germany

**Keywords:** genetics, environment, skeletal muscle development, skeleton, muscle

Skeletal muscle constitutes approximately 40% of body weight and serves as the primary source of animal protein for humans. As the largest organ in the body (1, 2), it is composed of multinucleated muscle fibers and mononuclear cells (e.g., FAPs, myogenic stem cells, and macrophages), making it a highly heterogeneous tissue. Skeletal muscle plays a pivotal role in movement, systemic metabolism, maintaining metabolic health in animals, enhancing meat production, and improving meat quality. In the pig industry, achieving optimal meat quality traits while ensuring carcass quality remains a significant challenge. Researchers have employed various sequencing methods, including transcriptomic analysis (mRNA, miRNA, lncRNA, and circRNA) and epigenetic studies (DNA methylation, m6A modification) (3–6), to regulate muscle growth-related genes and uncover novel regulatory factors associated with skeletal muscle development and meat quality phenotypes. Single-nucleus RNA sequencing (snRNA-seq) addresses the challenge of shared cytoplasm in multinucleated muscle fibers by isolating nuclear gene expression profiles, enabling the analysis of transcriptional heterogeneity (5). This technique facilitates the identification of over 10 distinct cell types (7) and highlights the adaptive advantages of multinucleated cells (8). Furthermore, snRNA-seq can distinguish muscle fiber types (I, IIa, IIb, etc.) and their intranuclear functional divisions, revealing dynamic changes in muscle nuclei (9). In animal breeding, leveraging single-cell sequencing to identify key cell subtypes and regulatory genes is an effective approach for enhancing skeletal muscle development and optimizing meat quality. Beyond genetics, environmental factors dynamically interact with skeletal muscle development via multi-dimensional mechanisms. Integrating multi-omics technologies to dissect the gene-environment interaction network offers new insights and potential targets for improving skeletal muscle development and meat quality.

This Research Topic aims to deepen our understanding of how genetic and environmental factors regulate skeletal muscle fiber growth and metabolism. This Research Topic is aimed at collecting papers suitable to improve our knowledge and understanding on identification of meat phenotypes in livestock and poultry, omics study on meat quality, differentiation of skeletal muscle, functional study of skeletal muscle exosomes, types and transformations of muscle fibers, metabolic and endocrine control of muscle development and meat science, and other relevant aspects of skeletal muscle and meat science.

This Research Topic includes 7 papers covering the aforementioned aspects. Six of them focus on the mechanisms of muscle fiber development, metabolism, and the regulation of skeletal muscle development by nutritional and additive components. Specific contents are as follows:

Zhang et al. analyzed the phenotypic and transcriptomic features of pectoralis muscles in male and female black Muscovy ducks at 28, 42, and 70 days post-hatching, revealing the molecular mechanisms of muscle development across growth stages. Zhao et al. identified the low-energy protein feed with the lowest cost by evaluating growth performance, baking loss, and taste score as key indicators. Li et al. demonstrated that increasing the slaughter weight to 125 kilograms or higher decreases the lean meat percentage in live talent meat pigs and does not enhance the quality attributes of pork. Muscle fiber development and characteristics in broilers are key factors affecting growth performance and essential for producing high-quality chicken meat. Guanidinoacetic acid (GAA) is a critical endogenous substance in animal creatine synthesis. Hong et al. used multi-omics analysis to investigate the regulatory effects of different GAA levels on broiler muscle development and its molecular mechanisms. Mullenix et al. identified a novel feed additive, microencapsulated NUQO© NEX (a combination of plant and algal extracts), which improved broiler fattening performance parameters, overall feed conversion rate, processed carcass weight, and breast meat slice yellowness at varying inclusion levels. Song et al. revealed defects in lipid metabolism and insulin signaling in low birth weight goats via transcriptome analysis. These goats may accumulate lipids in skeletal muscle by disrupting liver insulin function.

In conclusion, the aforementioned research results have provided substantial new data on the genetic and nutritional regulation of skeletal muscle development. Despite the extensive literature and evidence available on this critical topic, the papers published in this topic highlight that many aspects of skeletal muscle development remain to be elucidated, particularly regarding its heterogeneity and the transcriptional and metabolic regulation during growth. Future studies will leverage advanced bioinformatics techniques to comprehensively unravel the complexity and systematic nature of skeletal muscle development.
